# Recent Progress in Sensing Technology Based on Triboelectric Nanogenerators in Dynamic Behaviors

**DOI:** 10.3390/s22134837

**Published:** 2022-06-26

**Authors:** Linjie Yao, He Zhang, Jiqing Jiang, Zhicheng Zhang, Xianglong Zheng

**Affiliations:** 1 College of Civil Engineering & Architecture, Zhejiang University, 866 Yuhangtang Road, Hangzhou 310058, China; 3160101475@zju.edu.cn (L.Y.); zjuzhanghe@zju.edu.cn (H.Z.); jszzc@zju.edu.cn (Z.Z.); 2 Center for Balance Architecture, Zhejiang University, 148 Tianmushan Road, Hangzhou 310061, China; 3 Department of Civil Engineering, Zhejiang University City College, 51 Huzhou Street, Hangzhou 310015, China; jiangjq@zucc.edu.cn; 4 The Architectural Design & Research Institute of Zhejiang University, 148 Tianmushan Road, Hangzhou 310061, China

**Keywords:** triboelectric nanogenerator, dynamic behaviors, translational motion, rotational motion, pressure sensor

## Abstract

Under the trend of the rapid development of the internet of things (IoT), sensing for dynamic behaviors is widely needed in many fields such as traffic management, industrial production, medical treatment, building health monitoring, etc. Due to the feature of power supply independence and excellent working performance under a low-frequency environment, triboelectric nanogenerators (TENGs) as sensors are attracting more and more attention. In this paper, a comprehensive review focusing on the recent advance of TENGs as sensors for dynamic behaviors is conducted. The structure and material are two major factors affecting the performance of sensors. Different structure designs are proposed to make the sensor suitable for different sensing occasions and improve the working performance of the sensors. As for materials, new materials with stronger abilities to gain or lose electrons are fabricated to obtain higher surface charge density. Improving the surface roughness of material by surface engineering techniques is another strategy to improve the output performance of TENG. Based on the advancement of TENG structures and materials, plenty of applications of TENG-based sensors have been developed such as city traffic management, human–computer interaction, health monitoring of infrastructure, etc. It is believed that TENG-based sensors will be gradually commercialized and become the mainstream sensors for dynamic sensing.

## 1. Introduction

With rapid urbanization, modern cities are encountering increasing problems and challenges, such as traffic congestion and infrastructure deterioration. Moreover, cities are constantly threatened by natural disasters such as floods, earthquakes, and typhoons. As a major trend, the smart city is believed to be a feasible way to solve these problems and improve the resilience of cities against natural disasters. Smart city construction requires comprehensive sensing and ubiquitous interconnection to provide information support. For example, the dynamic behaviors of infrastructure may reflect the structural operation states and are useful in decision making, risk prevention, and emergency response; vehicle motion data can be used for autonomous driving and traffic management. The dynamic behaviors are usually described by indexes including displacement, velocity, acceleration, frequency, pressure, etc. To monitor these indicators, plenty of technologies and sensors have been developed including optical fiber sensors [1,2,3,4], global positioning systems (GPS) based sensors [5,6,7,8], piezoelectric sensors [9,10,11], etc. However, most of these sensors are dependent on the external power supply, which causes inconvenience of maintenance and rising cost. For more convenient, intelligent, and environment-friendly requirements, the future sensors are better to be low-cost, self-powered, and easy to be maintained. Therefore, TENGs serving as novel self-powered sensors have attracted more and more attention. Similar to piezoelectric nanogenerators (PENGs) [12,13], TENGs can convert mechanical energy into electrical energy [14,15,16], which frees TENG-based sensors from dependence on the external power supply. The output power of TENG has reached 8.75 W/m^2^ [17]. The advantages of TENG over PENG are the relatively high voltage output and outstanding working performance in a low-frequency environment. Moreover, TENGs generate electricity output only under dynamic occasions, and the output characteristics are directly influenced by the external mechanical movement, which offers the possibility of TENGs for sensing dynamic behaviors. Some theoretical methods for the analysis and optimization of TENG have been proposed, which offer the theoretical basis for TENG sensors [18,19]. Recently, a theoretical model has been developed to model the non-planar elementary geometric TENGs such as cones, arcs, disks, etc. [19].

In recent years, great efforts have been made in device structures and materials to improve the performance and develop new applications of TENG sensors. In structures, various structures for different applications are developed based on four basic modes [20,21,22,23]. Additionally, combining other power generation principles (e.g., electromagnetic [24] and piezoelectric [25]) with triboelectricity through structural design is an effective method to improve device performance and broaden the sensing range. For some functional requirements, several special structures are developed such as the sphere [26], fiber [27], and rotation structures [28]. In terms of materials, the material selection of TENG is wider than that of most sensors because almost every material is of triboelectric effect. Different materials such as polymers [29], conductive oxides [30], and carbon-based materials [27] are applied for triboelectric layers and electrodes. Further, to improve the output performance, micro/nano structures are introduced to triboelectric materials through different methods of surface engineering. Technologies such as water-assisted oxidation (WAO) [31], electrospinning [29], and inductively coupled plasma (ICP) etching [32] are utilized to fabricate rough textures on material surfaces. Mold pouring [30] and photolithography [33] are used to form regular patterns on material surfaces. Based on the advance in structures and materials, TENG-based sensors for dynamic behaviors are applied in many fields such as dynamic response monitoring of civil structures and marine structures [34,35], motion state sensing in machinery [36], vehicle monitoring for traffic management [37], and human health monitoring [38], etc. To implement these applications, TENG-based sensing technologies for translational motion, rotational motion, and pressure are developed.

In this paper, we primarily focus on the recent progress in TENG as self-powered active sensors for dynamic sensing. In Section 2, structural designs of sensors based on four basic modes, hybrid nanogenerators, and several special structures will be introduced first. Then, material selection and optimization methods are presented in Section 3. Further, we introduce sensing technologies of TENGs applied in different fields in Section 4. Finally, we summarize the recent advances of TENGs as self-powered sensors and offer some prospects in Section 5.

## 2. Structural Design of TENGs with Different Modes

The structural designs of TENGs can be divided into three categories according to different modes. The four basic modes of TENGs are different in structures, thus demanding various structural designs. Moreover, the multi-mode TENGs can effectively improve the device performance by integrating the triboelectric device with electromagnetic or piezoelectric mechanisms. Thirdly, some special structures have also been introduced in the design of TENG, such as the sphere, fiber, and rotation structures.

### 2.1. TENGs with Four Basic Modes

**Figure 1:**
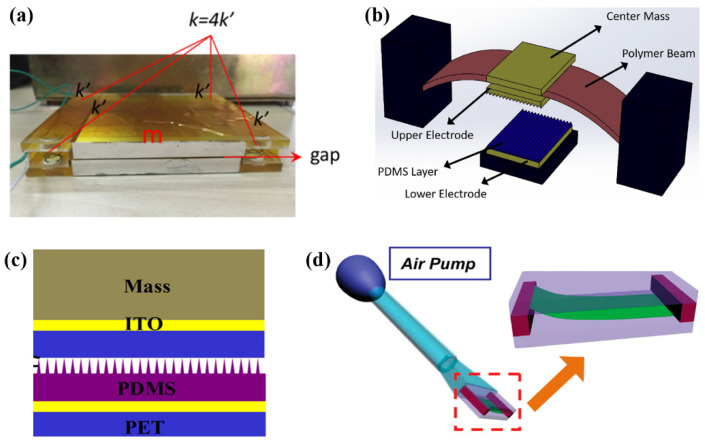
Structures of contact mode TENGs: (**a**) Spring-supported TENG (Reproduced with permission [20]. Copyright 2017 Elsevier); (**b**) An elastic beam-based TENG with two steady states (Reproduced with permission [41]. Copyright 2018 Elsevier); (**c**) A TENG with micro-pyramids on tribo-pair (Reproduced with permission [30]. Copyright 2016 Elsevier); (**d**) A TENG with flexible PTFE film (Reproduced with permission [43]. Copyright 2014 American Chemical Society).

As it is widely known, contact mode, sliding mode, single-electrode mode, and freestanding triboelectric layer mode are four basic modes of TENGs, each of which have typical structural features. In contact mode TENGs, two friction layers are placed face to face with an air gap between them. The mechanical energy is converted into electrical energy in the process of contact and separation of the tribo-pair. Since the contact mode is first proposed and the structure is relatively simple, plenty of sensors based on the contact mode have been reported [20,39,40,41]. In contact mode, springs are usually adopted to rebound and support the tribo-pair [20,41]. As the vibration accelerometer in Figure 1a, the two substrates are linked by four springs with an air gap and two friction layers attached to the opposite sides of the substrates [20]. When the springs are compressed and stretched due to the vibration of the host, the springs provide the forces that promote the substrate back to its original position. The stiffness of the spring and the size of the air gap are two important design parameters that directly affect the device’s sensitivity to vibration. In another contact mode TENG, the PET housing with an arc-shaped structure acts as the spring, providing the restoring force of tribo-pair [42]. As an alternative of spring, the elastic beam is used as the elastic support for a threshold-triggered shock sensor (Figure 1b) [41]. There are two steady states for the elastic beam: upper state and lower state. Initially, the beam holds the upper electrode and is at an upper steady position. Only when the shock acceleration exceeds a threshold, the beam will transfer from the upper state to the lower state, thus resulting in the contact between the PDMS layer and the upper electrode. Additionally, Xiang et al. fabricated an acceleration sensor that has micro-pyramids on the surface of the PDMS layer (Figure 1c) [30]. In this sensor, a mass is used to drive the movement of a triboelectric layer and the contact-separation process is realized by the change in contact area caused by the deformation of the pyramids. Thus, the device does not require any additive elastic component nor much space for the contact-separation process. In another TENG sensor, both ends of a flexible PTFE film are fixed on two spacers, respectively, and the PTFE film is placed between two electrodes (Figure 1d) [43]. When air flows through the PTFE film, it will vibrate to contact with the two electrodes, resulting in power output.

**Figure 2:**
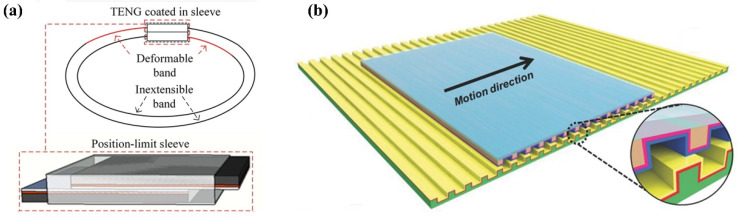
Structures of sliding mode TENGs: (**a**) A wearable TENG integrated into a deformable band (Reproduced with permission [21]. Copyright 2019 The Authors); (**b**) A TENG with grating structures (Reproduced with permission [33]. Copyright 2019 WILEY-VCH Verlag GmbH & Co., KGaA, Weinheim, Germany).

Different from the contact mode TENG, the two friction layers in sliding mode always keep close contact and move laterally. Due to the feature of lateral sliding, sliding mode-based sensors are often made into the film-like structure which is flexible and is suitable for the application of wearable sensors. For example, a wearable respiration sensor is fabricated based on sliding mode [21]. As shown in Figure 2a, the two triboelectric layers are coated in a sleeve to assure good contact between them [21]. Two ends of a wearable belt are linked to the two layers, respectively. Thus, the change in the circumference of the belt during the respiration process could drive the tribo-pair to slide relatively. Additionally, Wang et al. proposed a film-like TENG based on kinesio tapes [44]. The device is multilayer in which the tribo-pair is sandwiched by two layers of tapes with high stretchability and thin thickness. The thin and flexible feature enables the device to fit irregular surface shapes. The opposite ends of the two friction layers are stuck to the corresponding tapes. When the device is stretched and released, the two friction layers will slide relatively. The sliding mode TENGs can also be fabricated with multiple friction interfaces for better device performance [45]. Instead of wearable devices, sliding mode TENGs are also used to detect translational motion through the design of grating structures. As illustrated in Figure 2b, there are two micro gratings with a period of 200 μm on the surfaces of the moving part and static part, respectively [33]. Due to the design of the grating, even if the relative movement of the friction pair is non-reciprocating, the generated signal is still an AC output containing movement information. The period of grating determines the detection resolution, that is, the smaller period corresponds to higher detection resolution.

**Figure 3:**
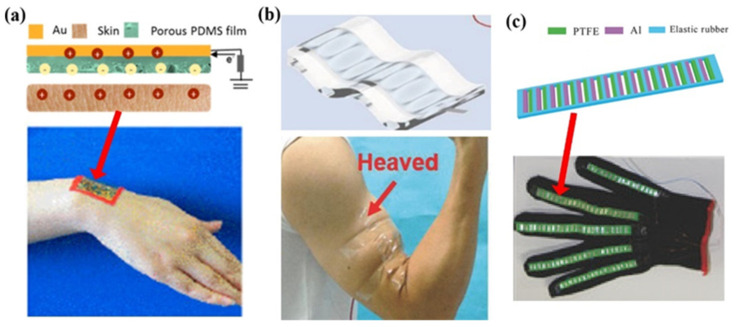
Structures of single-electrode mode TENGs: (**a**) A flexible film-structured TENG attached to skin (Reproduced with permission [22]. Copyright 2018 American Chemical Society); (**b**) A flexible and stretchable TENG with wrinkled structure (Reproduced with permission [46]. Copyright 2018 WILEY-VCH Verlag GmbH & Co., KGaA, Weinheim, Germany); (**c**) A ladder-structured TENG integrated into a smart glove (Reproduced with permission [47]. Copyright 2018 WILEY-VCH Verlag GmbH & Co., KGaA, Weinheim, Germany).

In single-electrode (SE) mode, a pure dielectric layer serves as one friction layer, with another friction layer being a conductor as the electrode or a composite layer consisting of dielectric material and electrode. The SE mode TENG is especially suitable for the wearable sensor in which skin may act as one of the friction layers. As shown in Figure 3a, by taking advantage of the skin as one friction layer, the sensor could be designed as a flexible film with a small thickness [22] and used on the wrist or ankle for motion sensing (Figure 3a). However, the device in [22] is flexible but not stretchable, and therefore, the size of the device should be small enough, otherwise, the comfortability of the device will be influenced. To deform simultaneously with skin, the sensor should be of both good stretchability and flexibility. Zhan et al. fabricated a wrinkled TENG as a stretchable motion sensor [46]. The sensor is made of a PDMS plate as friction material and a transparent electrode (Figure 3b). There are wrinkles on the surface when the sensor is loose, and the sensor will become smooth when stretched, which is very similar to human skin. Owing to the wrinkled structure, the sensor is of good stretchability to deform with skin even when mounted on the heaved muscle of the arm (Figure 3b). The SE mode TENG is also used in smart gloves rather than skin [47]. In the device in Figure 3c, there is a stretchable elastic rubber as the substrate with a ladder structure, and several small-sized TENG units are embedded in the elastic rubber to form an array. Due to this ladder structure design, the TENG exhibits excellent stretchable and flexible performance in process of motion sensing. Although SE mode TENG shows an advantage with wearable sensors, its charge transfer efficiency can only reach 50% due to the electrostatic shield effect, while those of paired-electrode devices could reach nearly 100% [48].

**Figure 4:**
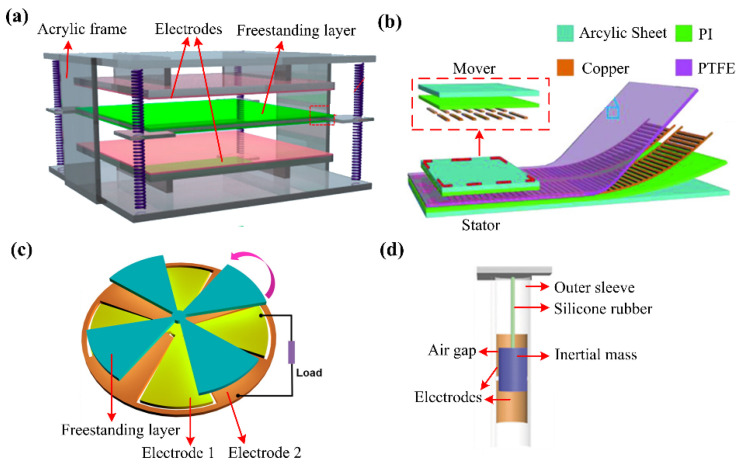
Structures of freestanding triboelectric-layer-based TENGs: (**a**) An FTENG with contact mode for vibration sensing (Reproduced with permission [23]. Copyright 2014 American Chemical Society); (**b**) A FTENG with sliding mode for translational motion sensing (Reproduced with permission [51]. Copyright 2018 WILEY-VCH Verlag GmbH & Co., KGaA, Weinheim, Germany); (**c**) A non-contact TENG with a disk-like structure (Reproduced with permission [52]. Copyright 2014 American Chemical Society); (**d**) A non-contact TENG with a tube-like structure (Reproduced with permission [53]. Copyright 2017 WILEY-VCH Verlag GmbH & Co., KGaA, Weinheim, Germany).

To prevent the electrostatic shield effect, the freestanding triboelectric-layer-based nanogenerator (FTENG) is proposed with the paired-electrode structure [49]. In FTENG with contact mode (CFTENG), the freestanding layer is placed between the two opposite electrodes. In the CFTENG in Figure 4a, two Al electrodes are fixed in an acrylic frame with an FEP layer (the freestanding layer) supported by springs [23]. Compared with CFTENG, the FTENG with sliding mode (SFTENG) is more widely used with different structural designs. Firstly, SFTENG could be designed with a structure composed of a mover and a stator [50,51]. As the freestanding layer, the mover does not necessarily have to be attached with an electrode and a lead wire, and it could be fixed on moving objects such as humans and vehicles to monitor their motions [49]. As shown in Figure 4b, the mover is composed of two columns of copper strip coated on a polyimide (PI) film which is attached to an acrylic sheet, while the stator is composed of a PTFE film as the dielectric layer, and two interdigitated electrodes with a PI and an acrylic sheet as the base [51]. The interdigitated electrodes can lead to an alternating output when the mover slides linearly on the stator. Secondly, SFTENG could be designed with a non-contact structure, in which there is an air gap between the tribo-pair during the working process, to prevent the energy loss from the friction. Based on the non-contact structure, Lin et al. proposed a disk-like TENG as shown in Figure 4c [52]. The two electrodes of the device are fixed on the stationary base with the electricity generated in the rotation process of the freestanding layer. Yu et al. proposed a tube-like TENG which is of a larger specific sensing area compared with planar devices [53]. As shown in Figure 4d, the device is composed of an outer sleeve tube coated with two Cu electrodes and an inner cylindrical inertial mass suspended by highly stretchable silicone rubber. They found that the output of the device decreases with the increase of the gap, which is controlled at 2 mm to obtain the best performance.

### 2.2. Structural Design of TENG Sensors with Multi-Mode

To improve the performance of the TENG, different generators including electromagnetic (EMG) and piezoelectric generators (PENG) are introduced and integrated into TENG to form new hybrid generators.

**Figure 5:**
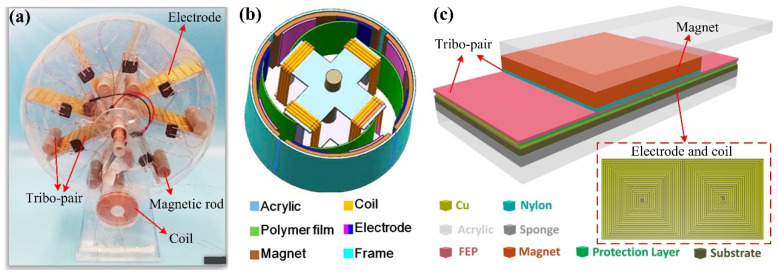
Structures of triboelectric-electromagnetic hybrid generators: (**a**) A waterwheel-like structure with EMG acting as the base (Reproduced with permission [24]. Copyright 2018 Elsevier); (**b**) A wind speed sensor with EMG acting as the base (Reproduced with permission [54]. Copyright 2018 American Chemical Society); (**c**) A SFTENG with EMG integrated into the structure (Reproduced with permission [55]. Copyright 2016 American Chemical Society).

In triboelectric-electromagnetic hybrid generators, there are primarily two design strategies to realize the combination. In the first one, the rotator-stator structure of the EMG is treated as the base, with the tribo-pair of TENG integrated into the structure. As shown in Figure 5a, the device is a waterwheel-like structure with coils as the stator and several magnetic rods as the rotator [24]. The output of the EMG is generated due to the rotational motion of the magnet rods, which causes alternating magnetic flux in coils. One friction layer of TENG is attached to wrap around the magnetic rod, while several electrodes are fixed on the acrylic substrate as another friction layer with one rod between two electrodes. When the rods rotate as the rotator of EMG, the friction occurs between the magnet rods and the electrodes simultaneously. Therefore, the EMG and TENG both generate electricity during the rotation of the rotator. In addition, a similar structure is also used as a wind speed sensor (Figure 5b) [54]. In this device, the rotator is composed of a shaft and a cross-shaped vertical structure with four groups of coils, while the stator is composed of a cylindrical acrylic with four pieces of arc-shaped magnets attached to the inner wall [54]. When the rotator is driven by the wind, the coils cut the magnetic induction lines to produce electricity. The tribo-pair of TENG is composed of electrodes attached to the magnet and four elastic blades fixed on the rotator. The friction between the elastic blades and the electrodes is induced simultaneously by the rotation of the rotator.

As another strategy, the TENG is utilized as the base with the EMG integrated into the structure. As shown in Figure 5c, the typical structure of SFTENG is adopted in this device [55]. The magnet of the EMG is integrated into the freestanding layer, while the coils of the EMG unit are embedded in electrodes of the TENG. Therefore, the relative movement of the tribo-pair causes both the friction between the tribo-pair and the variation of the magnetic flux in coils. The combination of triboelectric and electromagnetic brings some advantages to the sensor. The sensing frequency range of the device could be broadened because the TENG component performs better due to excitations with lower frequencies while the EMG components are more efficient by excitations in higher frequencies [24,54]. Additionally, higher sensing sensitivity could be obtained by utilizing the high output voltage of TENG and the high output current of EMG [55,56].

**Figure 6:**
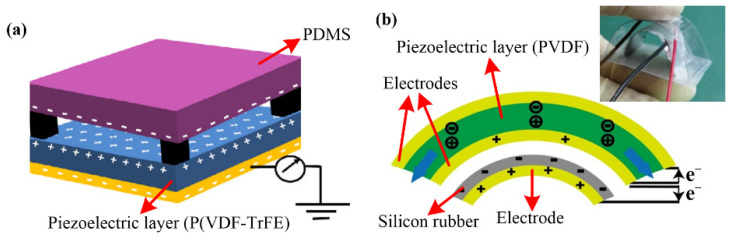
Structures of triboelectric-piezoelectric hybrid generators: (**a**) A pressure sensor with a self-poled P(VDF-TrFE) sponge acting as the tribo-layer (Reproduced with permission [25]. Copyright 2017 Tsinghua University Press and Springer-Verlag GmbH Germany); (**b**) A vibration sensor with a double-arched structure (Reproduced with permission [57]. Copyright 2017 Elsevier).

In triboelectric-piezoelectric hybrid generators, the TENG device is usually employed as the base with PENG integrated into the structure. The piezoelectric layer could be used as the tribo-layer directly or embedded in the tribo-layer as another generator combined with TENG. In a pressure sensor, a self-poled P(VDF-TrFE) sponge is applied as the positive tribo-layer of the TENG (Figure 6a) [25]. When mechanical pressure is exerted on the device, the piezoelectric potential difference caused by the piezoelectric effect will contribute some extra surface charges, thus increasing the surface charge density and further enhancing the output performance. Specifically, the sensing range of this sensor is broadened from 0.05–50 kPa to 0.05–600 kPa by the hybrid mechanism of triboelectricity and piezoelectricity [25]. When the piezoelectric layer is embedded in the tribo-pair as a separate generator, the TENG and PENG components will work simultaneously [26,57]. In a vibration sensor, the tribo-pair is designed as a double-arched structure (Figure 6b), which provides natural resilience to the tribo-pair during the contact and separation process [57]. In the upper layer of the tribo-pair, the piezoelectric layer (PVDF) is sandwiched by two Al electrodes to form a composite triboelectric-piezoelectric layer. The electrode on the inner surface of PVDF is shared by both the piezoelectric and triboelectric units. The charges generated by the two generators are superimposed on the shared electrode, which enhances not only the output of every single generator but also the total output energy.

### 2.3. Other Special Structural Designs

**Figure 7:**
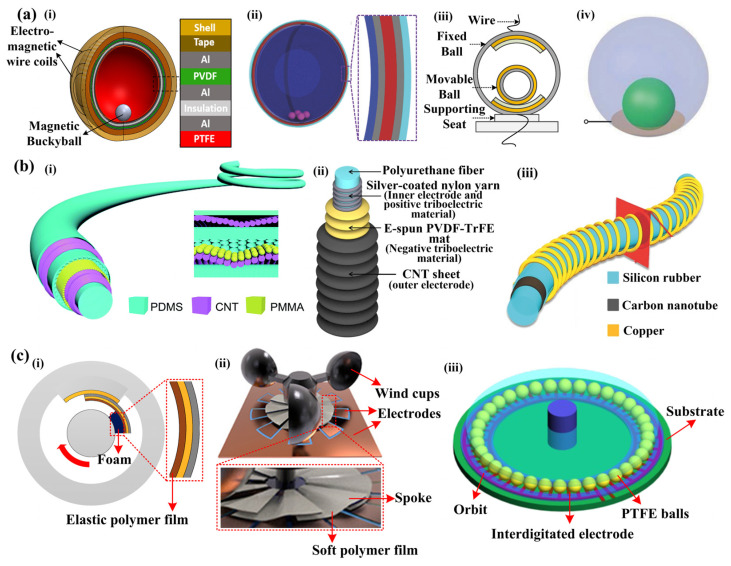
Structures of (**a**) (**i**–**iv**) sphere-based sensors, ((**i**) Reproduced with permission [26]. Copyright 2018 Elsevier; (**ii**) Reproduced with permission [59]. Copyright 2017 WILEY-VCH Verlag GmbH & Co., KGaA, Weinheim, Germany; (**iii**) Reproduced with permission [58]. Copyright 2020 The authors; (**iv**) Copyright 2013 WILEY-VCH Verlag GmbH & Co., KGaA, Weinheim, Germany; (**b**) (**i**–**iii**) fiber-based sensors, (**i**) Reproduced with permission [27]. Copyright 2017 The Royal Society of Chemistry; (**ii**) Reproduced with permission [63]. Copyright 2016 The authors; (**iii**) Reproduced with permission [64]. Copyright 2016 WILEY-VCH Verlag GmbH & Co., KGaA, Weinheim, Germany and (**c**) (**i**–**iii**) rotator-stator structured sensors. ((**i**) Reproduced with permission [28]. Copyright 2019 The authors; (**ii**) Reproduced with permission [65]. Copyright 2018 American Chemical Society; (**iii**) Reproduced with permission [66]. Copyright 2016 IOP Publishing Ltd. (Bristol, UK)).

In addition to the four basic modes and hybrid generators introduced above, there are several other typical structures such as the sphere, fiber, and rotation structures, etc. Some typical sphere-based sensors are illustrated in Figure 7a [26,58,59,60]. In these devices, there is a spherical shell and a cavity inside, with an electrode and friction layer adhered to the inner surface of the spherical shell. Therefore, the spherical shell is often of a multilayer structure (Figure 7(ai,aii)). Moveable objects are placed in the cavity as another friction material. In most cases, the moveable objects are also spherical such as magnetic buckyballs [26], steel balls [59,61], acrylic balls covered with copper foil [58], and PFA spheres [60] (Figure 7(ai–aiv)). Once the shell is designed impermeable, the liquid could be employed as the friction material [62]. The moveable objects in the spherical shell could return to the original position due to their weight instead of springs, to reduce the use of mechanical components and further promote the device’s reliability [58]. Due to the 3D symmetrical structure, the sphere-based TENG has a unique advantage for sensing and energy harvesting in multi-directions.

The second typical structure is fiber. In fiber-based TENG, the tribo-pair is integrated into a slender and long fiber. There is a core fiber taken as the substrate with the electrode and dielectric material wrapped layer by layer from inside to outside, forming a coaxial fiber (Figure 7b) [27,63,64]. The fiber-based TENG could be designed as highly stretchable. Sim et al. proposed a stretchable triboelectric fiber that can be stretched up to 50% strain (Figure 7(bii)) [63]. The electrode could be stretched together with the core fiber due to its fabrication by twining silver-coated nylon yarn around the core fiber. The PVDF-TrFE/CNT shell is also stretchable due to its wrinkle structure. In another stretchable device, there is a stretchable core fiber made from silicon rubber, an inner stretchable electrode made from conductive CNT/polymer, and an outer stretchable electrode made of a copper coil (Figure 7(biii)) [64]. Therefore, a strain of up to 70% could be realized on this device. In another fiber-like TENG, the steel spring is inserted into a hollow latex tube to provide the stretchability and to act as the electrode [35]. In general, fiber is a highly integrated structure with advances in flexibility, lightweightness, and thinness (e.g., the diameter of 3 mm [67], 500μm [68], and 490 μm [63]), thereby, fiber-based TENGs even could be weaved into smart cloth to sense human motion.

The rotator-stator structure is another special structural design in which the tribo-pair is integrated into the rotator and stator, respectively [65,66]. The rotator rotates relative to the stator, causing friction between the tribo-pair and triggering alternate electrical output. The hard friction will cause large friction force and material wear. To achieve lower frictional resistance and better durability, soft sliding friction is realized instead of hard friction in several devices. In a rotational speed sensor, the soft friction is realized by utilizing an elastic polymer film and a foam (Figure 7(ci)) [28]. The polymer film is of relatively good elasticity to support the friction layer, ensuring the contact area of the tribo-pair, while the foam could be compressed and deformed to cushion the friction process. In another wind speed sensor, soft polymer films are adopted to realize the soft sliding friction (Figure 7(cii)) [65]. One side of the soft film is fixed on the spoke of the rotator, while the other side is in contact with the stator to realize the soft friction. Although soft friction reduces frictional resistance to a certain extent, the friction is still relatively large. Therefore, the rolling friction is introduced to further reduce the frictional resistance. In a ball-bearing structured TENG [66], there is a disk-like substrate coated with interdigitated electrodes, while two rings (inner ring and outer ring) are pasted on the substrate serving as the orbit of the balls (Figure 7(ciii)) [66]. When the balls roll along the orbit, alternate electricity will be generated due to the design of interdigitated electrodes.

## 3. Materials for TENG Sensors

Tribo-pair and electrodes are two indispensable parts of all kinds of TENGs, and the materials of them will influence the output performance of TENGs. Plenty of materials and technologies have been developed and applied to TENG sensors.

### 3.1. Materials for Tribo-Pair

Materials for tribo-pair directly affect the device output due to the difference in triboelectric characteristics. For materials of tribo-pair, their triboelectric characteristics are the most important influencing factors. To further enhance the output of the device, different technologies of surface engineering have been applied to the tribo-pair or electrodes.

#### 3.1.1. Materials for Tribo-Pair

The influence of tribo-pair on the performance of TENG devices is mainly determined by surface charge density. According to the theoretical models of TENG, the surface charge density of dielectric material shows a linear relationship between the transferred charge and output voltage [69]. The surface charge density of the material in the tribo-pair is mainly determined by the ability to gain or lose electrons. The materials with strong electron affinities tend to attract a larger number of electrodes, while materials with strong electro-positivity are prone to lose electrons. The difference in charge densities of the tribo-pair should better be larger for higher output. To quantitatively evaluate the triboelectric characteristics, the triboelectric charge densities (TECD) versus liquid mercury of different materials are tested to obtain a quantitative triboelectric series (Table 1) [70].

**Figure 8:**
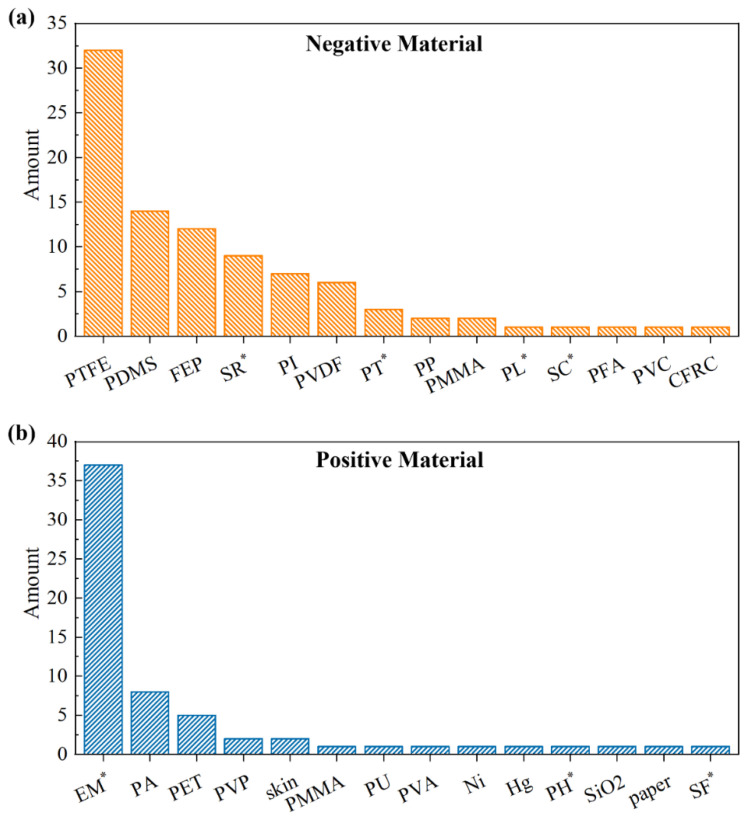
The statistics histogram of (**a**) negative materials of tribo-pair, and (**b**) positive materials of tribo-pair. * represents the abbreviation: SR (silicone rubber); PT (P(VDF-TrFE)); PL (Parylene); SC (silicone); EM (electrode material); PH (PDMS/PVDF-HFP); SF (silk-fibroin).

The materials close to the top of Table 1 are suitable for negative tribo-layer, while those close to the bottom are suitable for positive tribo-layer. To clearly show the usage of various materials in TENG sensors, a statistic is conducted and the results are illustrated in Figure 8. The majority of negative triboelectric materials are polymer materials (Figure 8a). Among these materials, PTFE, PVDF, and FEP are of strong electron affinities due to the component of fluorine, which is of the strongest electro-negativity among all elements. PTFE is the most popular negative triboelectric material due to its properties of excellent electrical insulation, high chemical stability, high age resistance, and low friction coefficient (Figure 8a). Besides a well-known piezoelectric material, PVDF is also a well-behaved triboelectric material [71,72]. Li et al. compared the output of PET-, PI-, and PVDF-based TENGs, and found the PVDF-based TENG with the highest short-circuit current and open-circuit voltage [29]. Further, the ability to capture electrons of PVDF is enhanced with functional groups with large electro-negativity (e.g., -TrFE) grafted to PVDF [63,73]. Compared with the above-mentioned materials, PDMS is widely used in impact sensors [74,75] and stretchable and transparent devices [76,77] because of its advantages in low elastic modulus, transparency, and stretchability. In addition to the triboelectric material, PMMA is often used as the base of the device due to its high transparency and high mechanical strength [78].

Metals and polymers are two typical kinds of materials used for positive tribo-layer. As shown in Figure 8b, metals account for a large proportion of positive materials because most metals are prone to losing electrons. Metals such as Al [32], Cu [79], and Ni [80] are widely used. In these cases, most metals play the dual role of electrodes and tribo-pair, which is a common practice of TENG sensors. Moreover, liquid metal such as Hg has also been adopted because liquid metal may achieve intimate contact with dielectric materials compared with solid metal, causing higher output performance [71]. As for polymers, the adopted materials include PA, PET, PMMA, and PU, etc. PA (nylon) is often selected as the positive tribo-layer due to its excellent abrasion resistance and electro-positivity [81]. PET is another positive material with high transparency. In a pressure sensor, PET and PDMS are chosen for the tribo-pair to fabricate a device with high transparency [77]. Through a special process, a PU foam is fabricated with a negative Poisson’s ratio and used in a strain sensor, in which the PU foam will expand in a direction perpendicular to the stretch, causing the contact of tribo-pair [82]. In addition to polymers and metals, there are several other options for positive materials including SiO_2_ [33], silk-fibroin [83], skin [84], and even common paper [85], which further underlines the diversity and flexibility of the choice of TENG’s materials.

#### 3.1.2. Surface Engineering of Tribo-Pair Material

In addition to the selection of materials, the surface topography of tribo-pair is also an important issue for device performance. Generally, the higher surface roughness of friction interface will result in higher surface charge density. Therefore, various irregular and regular micro/nano topographies are introduced on the material surface through surface engineering to roughen the surface.

For irregular micro/nano topography, technologies such as water-assisted oxidation (WAO), electrospinning, and inductively coupled plasma (ICP) etching, etc., are adopted to roughen the surface of tribo-pair. WAO is a simple and low-cost technology to create nanostructures on the surface of the metal. The whole process of WAO could be summarized as heating and oxidizing the metal in deionized water for a certain time. In a wind-driven TENG, nanograss-like structures are created on the surface of Al electrodes through the WAO process [31]. Additionally, the morphology of the nanostructure may vary with the type of metal. For Al, Cu, and Zn, the nanostructures are nanograss-like, cubic-like, and needle-like, respectively [86]. The WAO-treated Al is found to be the most appropriate choice due to its higher improvement in the output of TENG (79.7% compared with 42.6% of Cu) and shorter processing time (about 1 h compared with 12 h of Zn) [86].

**Figure 9:**
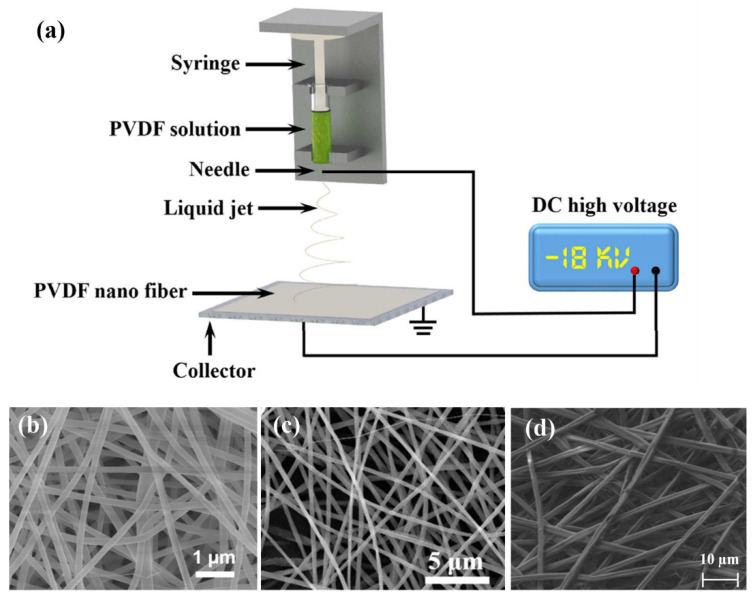
(**a**) The schematic diagram of the electrospinning process (Reproduced with permission [71]. Copyright 2017 American Chemical Society). The SEM image of nanofibers made of (**b**) nylon, (Reproduced with permission [29]. Copyright 2016 WILEY-VCH Verlag GmbH & Co., KGaA, Weinheim, Germany); (**c**) PVDF (Reproduced with permission [71]. Copyright 2017 American Chemical Society) and (**d**) PVP (Reproduced with permission [88]. Copyright 2018 Elsevier).

Electrospinning technology is very suitable for manufacturing nanofibers because of its low cost and simplicity. The schematic diagram of the electrospinning process is shown in Figure 9a [71]. The polymer solution is injected into a syringe, then the spinning is conducted in a strong electric field to fabricate nanofibers. The tribo-pair with nanofibers are of large specific surface area and considerable roughness [71,87,88]. For example, the SEM image of nanofibers made of nylon [29], PVDF [71], and PVP [88] in TENG-based sensors are illustrated in Figure 9b–d. The nanofiber fabricated by electrospinning are interlaced with each other thus contributing to a large specific surface area, and the average diameter of the nanofibers is small as 200 nm of nylon, 290 nm of PVDF, and 1500 nm of PVP. The nanofibers are effective in improving the output of TENGs. For instance, the open-circuit voltage of an electrospinning nanowire-based TENG reaches up to 1163 V, and the output current is increased by more than 4 times compared with that of TENG without nanowires [89].

**Figure 10:**
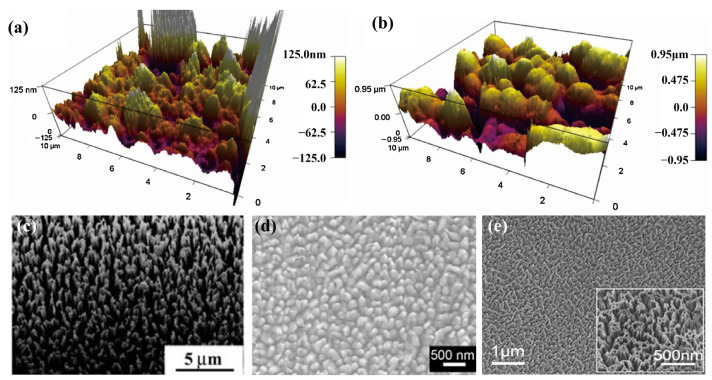
The AFM images of (**a**) ICP-treated and (**b**) non-treated PTFE films (Reproduced with permission [91]. Copyright 2018 Elsevier). Different nanostructures fabricated on the material surface: (**c**) nanowires (Reproduced with permission [32]. Copyright 2013 WILEY-VCH Verlag GmbH & Co., KGaA, Weinheim, Germany); (**d**) nanoparticles (Reproduced with permission [92]. Copyright 2013 American Chemical Society) and (**e**) nanorods (Reproduced with permission [93]. Copyright 2014 WILEY-VCH Verlag GmbH & Co., KGaA, Weinheim, Germany).

ICP etching is another method widely used to shape irregular topography on triboelectric materials [43,44,90]. In the ICP process, the sample is first placed in an ICP chamber, and then the reaction gases (O_2_, Ar, and CF_4_) are injected into the chamber and decomposed into plasma with strong chemical activity. The plasma flow passes through the surface of the material to etch the material through both chemical reaction and physical etching effect. In the atomic force microscopy (AFM) images of ICP-treated (Figure 10a) and non-treated (Figure 10b) PTFE films, the height of surface topographies are 960 nm and 120 nm, respectively [91], which proves that the surface roughness of the material is significantly improved after the ICP process. Different nanostructures such as nanowires (Figure 10c) [32], nanoparticles (Figure 10d) [92], and nanorods (Figure 10e) [93] are fabricated to improve the device performance. In the device with nanoparticles, the output voltage of the device reaches 1100 v [92]. Moreover, Zhu et al. found that the open-circuit voltage and short-circuit current of TENGs with nanostructures are about 4 times and 2 times that of TENGs without nanostructures, respectively [91].

**Figure 11:**
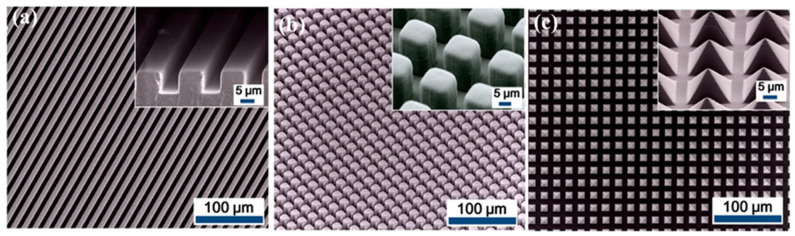
Regular structures on the material surface: (**a**) line-like structures; (**b**) cube-like structures, and (**c**) pyramid-like structures (Reproduced with permission [77]. Copyright 2012 American Chemical Society).

Regular structures on the material surface are of clearer shapes and more uniform arrangements. Generally, mold [73,74,94,95] and photolithography [33,52] are two methods utilized to create regular patterns. In the former method, polymer mixture is poured or spin-coated onto a mold with wanted concave patterns. The polymer film is peeled off after solidification to obtain a tribo-layer with regular structures. For example, nanopillar arrays are obtained by using anodic aluminum oxide templates and bring about an improvement of 110% on the output voltage [95]. Besides, Fan et al. compared the promotion effects of three regular structures including line-, cube-, and pyramid-like structures (Figure 11a–c), in which the device voltages are about 230% (line), 500% (cube), and 570% (pyramid) of that of non-treated TENG [77]. Further, a micro-frustum-arrays structure is developed and the device with this micro-structure shows a voltage exceeding 2 times that of the device with the micro-pyramid structure proposed in [77]. However, the fabrication of mold may increase the complexity of the process. In the latter methods, photolithography is utilized to directly engrave the designed patterns on the surface of tribo-pair to simplify the whole process. In a motion sensor, a glass plate as the slide layer is fabricated with a line-like structure through photolithography [33]. Moreover, Lin et al. employed the photolithography method to create microscale cubic structures onto the Al foil [52].

### 3.2. Materials for Electrodes

**Figure 12:**
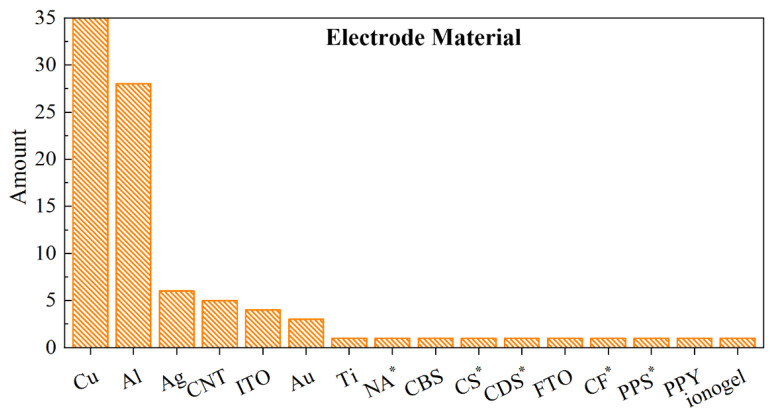
The statistics histogram of electrode materials. * represents the abbreviation: NA (Ni foam/Ag); CS (carbon sponge); CDS (conductive sponge); CF (conductive fabric); PPS (PEDOT:PPS).

For electrode materials, good conductivity is indispensable, while other physical properties such as transparency and flexibility are also significant in some specific cases. Materials for electrodes can be divided into metal and non-metal materials, of which metal materials account for the majority (Figure 12). The ideal metal for electrodes should be with low resistance, suitable surface topography, and low cost. Cu [56,64,79] and Al [24,90,96] are most widely used due to their good electrical conductivity and low cost. Moreover, metals such as Ag [44,63,97], Au [25,74,98], Ti [67], and Ni [97] have also been adopted. The device with Ag electrode is observed to have the highest output voltage compared with those of devices with Au-, ITO-, and Cu- electrodes because of the best electrical conductivity of Ag [80]. The Au-electrode possesses the smoothest surface topography due to its extremely high ductility [80].

However, for metal materials, the opacity and poor deformability have limited the feasibility of devices in some specific cases. Thus, some non-metal materials with electrical conductivity such as carbon-based materials, conductive oxides, and conductive polymers have also been developed as electrode materials. Carbon nanotube (CNT) and carbon nanofiber are often selected for flexible, stretchable electrodes due to the high conductivity, flexibility, and mechanical strength [66,68,99]. Carbon black-silicone rubber (CBS) is a kind of composite material fabricated by adding carbon black power into silicone rubber, making it both deformable as rubber and conductive as carbon [91]. The carbon sponge is also a viable choice as the electrode, in which the carbon fibers are utilized to construct a 3D skeleton with pores, offering considerable mechanical strength and light weight [29]. In conductive oxides, indium tin oxide (ITO) [30,77,83] and fluorine-doped tin oxide (FTO) are all utilized due to their features of transparency. In a pressure sensor, ITO is coated on PET film which shows transmittance of 85% in the visible and near-infrared region [77]. In conductive polymers, poly(3,4-ethylenedioxythiophene):poly(4-styrenesulfonate) (PEDOT:PSS) and polypyrrole (PPy) are employed due to the stretchability. The PEDOT:PSS electrode shows transparency of 90% and a maximum strain of 100% [46], while the PPy electrode-based TENG could be stretched with a strain up to 310% [76].

## 4. Applications of TENG-Based Sensors in Dynamic Behaviors

With rapid development of IOT, there are extensive demands for the sensing of dynamic behaviors in many fields such as dynamic response monitoring of civil structures, motion state sensing in machinery, vehicle monitoring for traffic management, and human health monitoring, etc. As self-powered devices, TENGs are of great application potential in these sensing fields. Sensing technologies of translational motion, rotational motion, and pressure are developed to realize these applications.

### 4.1. Sensing Technologies in Translational Motion

**Figure 13:**
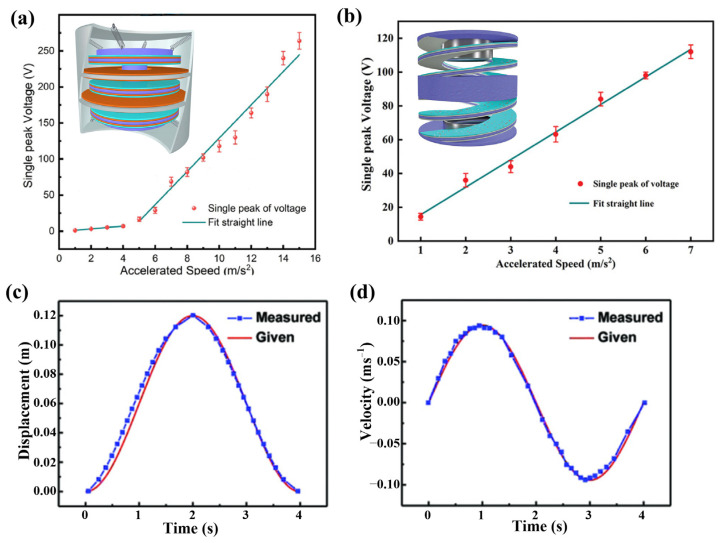
The acceleration-output relationships of (**a**) a multilayer suspension structured acceleration sensor (Reproduced with permission [100]. Copyright 2021 The authors) and (**b**) a double helix acceleration sensor (Reproduced with permission [101]. Copyright 2021 Wiley-VCH GmbH); (**c**) The real-time measured displacement and given displacement; (**d**) The real-time measured velocity and given velocity (Reproduced with permission [51]. Copyright 2018 WILEY-VCH Verlag GmbH & Co., KGaA, Weinheim, Germany).

Translational motion is common in life, such as the movement of the elevator, vehicles, pistons in the engine cylinder, and translational displacement due to the structure’s vibration. The translation motion can be described by indexes of displacement, velocity, acceleration, frequency, etc. The sensors are designed according to three different sensing mechanisms. The first kind is to calibrate the linear relationship between the peak value of electrical signals and the index to be tested. In the acceleration sensors in Figure 13a [100] and Figure 13b [101], the acceleration stimulated by the shaker is obtained according to the voltage peak. However, using this kind of sensor, only the peak value of the acceleration could be obtained with most of the information in the real-time responses lost.

To achieve real-time monitoring of the motion, a kind of grating structure (Figure 2b) [33] or interdigitated electrodes (Figure 4b) [51,102] are developed and applied in TENG. During the translational motion, every small tribo-pair on the comb finger or grating will generate a signal pulse when sliding overlap, which represents a tiny displacement increment of the moving object. Thus, we can also obtain the velocity according to the time intervals of the signal pulses. The smaller the size of gratings and interdigitated electrodes, the higher resolution of the displacement (Figure 13c) and velocity (Figure 13d) will be [51]. Zhou et al. proposed a motion sensor with micro-gratings, which realized an extremely small resolution of 173 nm for displacement and 1.2 μms^−1^ for velocity [33]. This mechanism is also used to sense the water/liquid level. Wang et al. proposed a non-contact TENG, in which several copper rings (electrodes) are attached to a tube with equally spaced arrangement [103]. Then the variation of the water level will be reflected by the electric pulses produced by the copper rings. A comprehensive sensing technique of water wave has also been developed to meet the need of ocean development and utilization. To quantitatively obtain the information of ocean waves, Zhang et al. developed a TENG-based sensor to obtain six basic ocean wave parameters including wave height, wave period, wave frequency, wave velocity, wavelength, and wave steepness [104].

Deriving the relationship between the dynamic behaviors and the detected electrical signals through theoretical models is the third strategy. In FTENG, there are two linear relationships between the mechanical indexes and the electrical outputs according to the mechanical-electric conversion theory of FTENG [23,105]. The first one is the linear relationship between the displacement of the freestanding layer and the open-circuit voltage (*V*_oc_). Another is the one between the instantaneous velocity of the freestanding layer and the short-circuit current (*I*_sc_). However, the linear relationships only exist in the condition of open-circuit or short circuit. In the latest study, Zhang et al. proposed a real-time sensing system based on TENG, in which the analytical relationship between the relative displacement of tribo-pair and the output voltage (voltage-transferred charge-displacement model) is derived [34]. Based on the theoretical model, the continuous and complete time-varying displacement is obtained with the detected signals. This work provides a useful and general tool for quantitively and continuously characterizing the dynamic behaviors in the long term.

**Figure 14:**
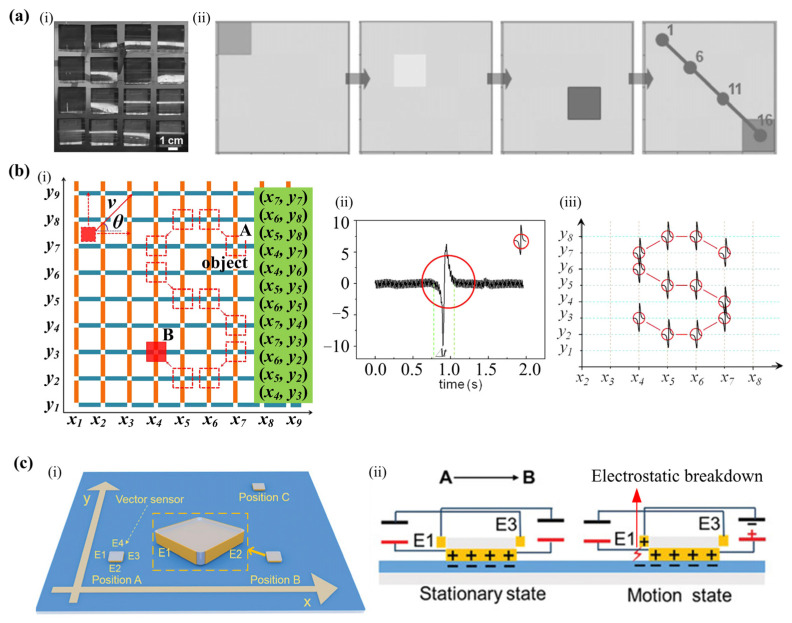
(**a**) (**i**) Image of the sensor array including 16 TENGs, (**ii**) the produced mapping images when an object moves along “1→6→11→16” (Reproduced with permission [96]. Copyright 2013 WILEY-VCH Verlag GmbH & Co., KGaA, Weinheim, Germany); (**b**) (**i**) The schematic diagram of trajectory tracking sensor array in which the trajectory of the object is from A to B, (**ii**) the current pulse signal from an output port versus time, (**iii**) the current pulse signal from both x and y (Reproduced with permission [107]. Copyright 2014 Elsevier); (**c**) (**i**) Schematic to describe the displacement of the motion vector sensor in a plane, (**ii**) working mechanism of the motion vector sensor (Reproduced with permission [108]. Copyright 2020 WILEY-VCH Verlag GmbH & Co., KGaA, Weinheim, Germany).

The trajectory tracking is usually realized by TENG arrays [96,106,107], in which each sensor generates a signal pulse when the object passes by. For example, Yang et al. fabricated a self-powered tracking system with a 4×4 matrix of TENG array as shown in Figure 14(ai) [96]. Figure 14(aii) is the mapping image that records the electric signals and marks the trajectory, which demonstrates clearly that the object is moving along the diagonal of the sensing array. To obtain a larger tracking range and better precision, the TENG arrays could be designed with a larger number of sensing units and a smaller size of sensors. In the trajectory tracking sensor array, sensors are assumed along the nodes in the sensing net [107]. The nodes on the same row or column share one metal strip as the electrode (Figure 14(bi)). When the object slides through a stripe, there will be an electric pulse in the corresponding x or y port (Figure 14(bii,biii)). Besides the TENG array, Yin et al. proposed a motion vector sensor (TVS) based on the contact-electrification effect and electrostatic breakdown [108]. As shown in Figure 14(ci), there are four charge collecting electrodes (E1–E4) fixed on the four sides of a substrate. The motion tracking of TVS can be displayed through the corresponding output signals of the four electrodes. For example, when the TVS slides from position A to position B on an FEP plane, electrostatic breakdown will occur between the FEP and the electrode at the back (E1), causing electric output in E1 (Figure 14(cii)).

### 4.2. Sensing Technologies in Rotational Motion

**Figure 15:**
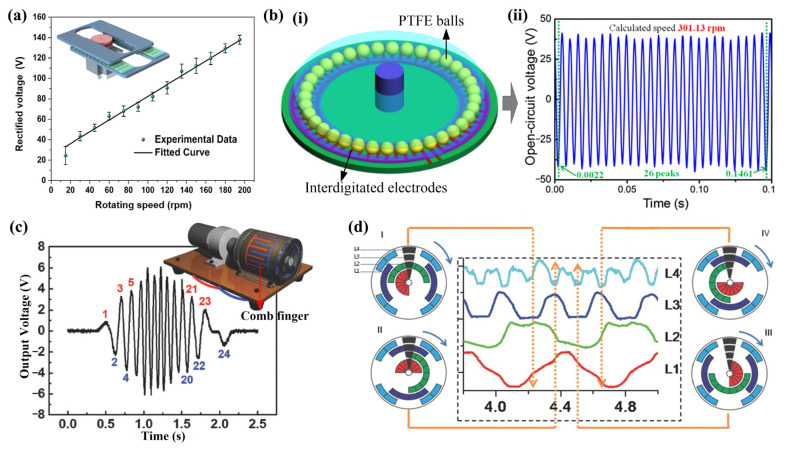
(**a**) The rotation sensor and its rectified voltage signals under different rotating speed (Reproduced with permission [109]. Copyright 2021 Elsevier); (**b**) (**i**) Schematic illustration of the ball-bearing structured TENG, (**ii**) the 26 voltage peaks at the rotating speed of 301 rpm, and the calculated speed of 301.13 rpm (Reproduced with permission [66]. Copyright 2016 IOP Publishing Ltd.); (**c**) A roller-bearing-based TENG for rotating sensing and its voltage in response to rotation movement with 360° rotation (Reproduced with permission [36]. Copyright 2018 WILEY-VCH Verlag GmbH & Co., KGaA, Weinheim, Germany); (**d**) The output voltage from four channels, which indicate that the rotator rotates to (I) 0°, (II) 90°, (III) 180°, and (IV) 270°. (Reproduced with permission [79]. Copyright 2015 WILEY-VCH Verlag GmbH & Co., KGaA, Weinheim, Germany).

Rotational motion widely exists in mechanical equipment, such as engines, downhole turbodrills, and bearings. The TENG sensors have been applied in monitoring rotating speed and angle on many occasions, such as steering wheels [36] and downhole turbodrills [28]. With the rotational motion of the host, the sensors will generate electric pulses. In sensing of rotating speed, the linear relationship between the voltage peak value and the rotating speed could be utilized (Figure 15a) [109]. However, the linear relationship is approximate and not stable. Calculating the rotational speed from the time interval between signal peaks is a more reliable means [66,110]. Li et al. proposed a ball-bearing structured TENG, in which the PTFE balls and interdigitated electrodes compose the tribo-pair (Figure 15(bi)) [66]. The sensor will generate a certain number of voltage peaks for each revolution of the bearing, which is used to calculate the rotating speed. As shown in Figure 15(bii), the sensed rotating speed (301.13 rpm) is very close to the actual speed (301 rpm) [66]. Through the sensing of rotating speed, the application of TENG as a self-powered sensor for the velocity of water flow detection is developed [111].

The sensing of the rotating angle could be realized with interdigital electrodes [36,112] or patterned electrodes [79]. Choi proposed an angle sensor, which contains two interdigitated electrodes and each electrode contains 12 comb fingers (Figure 15c) [36], which will produce 24 signal peaks, each representing 15° (=360°/24), in each circle of rotation. In terms of patterned electrodes, Wu et al. reported a TENG for angle measurement which consists of a rotator and a stator [79]. There are four channels on the rotator and several coded Cu electrodes on the stator (Figure 15d). The rotation angle of the rotator relative to the stator could be localized by the output signals from all four channels (L1–L4), in which the angular resolution of this sensor is 22.5° [79]. For this kind of sensor, the resolution is dependent on the number of channels or the fingers in the interdigitated electrodes.

### 4.3. Sensing Technologies in Pressure

The TENG-based pressure sensors have been applied in tactile sensing for human–computer interaction [77,98], human health monitoring for the heartbeat, respiration [38], pulse [113,114], and human joint movement [73,115], etc. For pressure sensors in different applications, the detection ranges are quite different. The detection ranges of pressure sensors applied in tactile sensing, monitoring for heartbeat, respiration, and pulse are usually less than 1500 Pa, while those of the human joint movement monitoring are from tens to hundreds of kPa. For the sensors which are used to detect pressure at a low level, the sensitivity and response speed is critical, especially when the pressure is very low (Table 2). In Table 2, one of the sensors is reported to realize the pressure sensing with a very low value of 0.23 Pa [113], which is even less than the contact pressure caused by a falling feather, about 0.4 Pa according to [77]. Additionally, most pressure sensors show a very short response time and recovery time of 100 ms and even shorter, which is critical in the application of real-time monitoring of fast-changing and time-varying pressure. In human joint movement, the pressures are at a higher level. Cao et al. reported a pressure sensor with a detection range of up to 200 kPa and realized the precise monitoring of gestures of finger joints [115]. To broaden the detection range, Yu et al. fabricated micro-frustum structure and nanofibers on PDMS films and finally realized the pressure detection up to 900 kPa [73], which was applied to elbows and distinguished the different bending angles of joints through the output voltage. In a recent study, the pressure sensor was also applied to identify the gender of the host and the joint type (knuckle, wrist, and elbow), which were judged by the output amplitude, response time, and recovery time [116]. The pressure sensor could also be applied to the traffic management system [37,117]. Through the TENG arrays, Yang et al. realized dynamic traffic monitoring including the detection of speed, overlapping, and overload [37]. Relying on the IoT platform, the proposed system can record traffic violations, warn of potential hazards, and assist traffic management through traffic data collection and timely analysis.

## 5. Conclusions and Perspectives

In this paper, we have reviewed recent advances in TENG as self-powered sensors for dynamic behaviors. The structures, materials, and applications of the sensors are introduced, which can be summarized as follows.

(1) To meet the application demands of TENGs in various fields, lots of structural designs of TENGs have been developed. Through special structural design, TENGs can be more effectively combined with the host to drive the relative motion of tribo-pair. Additionally, by combining the piezoelectric or magnetoelectric mechanism with the triboelectric mechanism through structural design, the output performance of the sensor will be improved and the detecting range will be broadened.

(2) The material design of TENG sensors mainly depends on the output performance of the device or the functional requirements of the applications on different occasions. The output performance of TENGs is affected by the intrinsic character of the tribo-pair materials for gaining or losing electrons. The larger the difference between the abilities of gaining or losing electrons, the higher the surface charge density will be. To obtain higher output performance, some special materials are applied. To meet the special needs of some applications such as wearable devices and human–computer interaction, some transparent, stretchable, or low-elastic modulus materials are applied in TENGs.

(3) The micro/nano structures on the tribo-pair surfaces can improve the output performance of TENGs by enhancing the ability to accommodate charges. The surface engineering technologies are applied to fabricate rough textures or regular patterns on the material surface, including electrospinning, ICP etching, photolithography, etc. The output performance of TENG sensors could be improved by the increase in the roughness of the tribo-pair surfaces.

(4) TENG-based sensors for dynamic behaviors are applied in many fields such as city traffic management, human–computer interaction, health monitoring of infrastructure, industrial machinery, the human body, etc. To realize quantitative sensing, the linear relationship between the peak values of electric output and the sensing indexes is utilized. However, only peak values of the indexes to be tested could be obtained, with most of the information in response time history lost. To realize real-time monitoring, grating structures and interdigitated electrodes are developed. For this kind of sensor, the sensing resolution depends on the size of gratings or interdigitated electrodes.

(5) The advances of TENG sensors in this review show great potential for TENG to be the mainstream of the next generation of sensors. To achieve a deeper understanding of the sensing mechanism and full-time domain sensing, the quantitative sensing techniques based on mechanical-electric conversion theory should be improved. Secondly, for better integration with precision equipment, the structures should be more compact and easier to manufacture. Thirdly, to improve the service stability, the durability of TENG sensors in the actual environment remains to be enhanced.

## Figures and Tables

**Table 1:** Part of the triboelectric series of materials and their triboelectric charge density (TECD) (Reproduced with permission [70]. Copyright 2019 The authors).

Materials	Abbr.	Average TECD (μC m^−2^)
Clear polyvinyl chloride	PVC	−117.53
Polytetrafluoroethylene	PTFE	−113.06
Polystyrene	PS	−103.48
Polydimethylsiloxane	PDMS	−102.05
Polyimide film	PI	−92.88
Polyvinylidene fluoride	PVDF	−87.35
Polyethylene	PE	−71.20
Clear cast acrylic	PMMA	−48.73
Silicone	-	−47.30
Polypropylene	PP	−27.23
Cast nylon 6	PA6	−18.35
Copy paper	-	−18.35

**Table 2:** The performance of TENG sensors for pressure.

Sensitivity	Detection Range	Response Time (ms)	Recovery Time (ms)	Reference
0.293 mV Pa^−1^	0.23 Pa~13.12 kPa	26	32	[113]
0.103 mV Pa^−1^	13.12~95.95 kPa			
0.21 Pa^−1^	20~1000 Pa	110	120	[98]
8.8 mV Pa^−1^	200~800 Pa	N/A	N/A	[88]
3.9 mV Pa^−1^	800~1400 Pa			
98.34 mV Pa^−1^	0~0.17 kPa	90	60	[118]
2.6 mV Pa^−1^	0.17~1.7 kPa			
0.13 mV Pa^−1^	1.7~30 kPa			
0.08 V kPa^−1^	2~60 kPa	200	35	[46]
0.008 V kPa^−1^	60~160 kPa			
2.67 kPa^−1^	0~4.7 kPa	62	52	[119]
0.46 kPa^−1^	4.7~20 kPa			
0.3 V kPa^−1^	0~200 kPa	N/A	N/A	[116]
0.05 V kPa^−1^	200~800 kPa			
2.97 V kPa^−1^	0~600 kPa	60	N/A	[73]
0.02 V kPa^−1^	600~900 kPa			

## Data Availability

Not applicable.
